# Knowledge and Antibiotics Prescription Pattern among Ugandan Oral Health Care Providers: A Cross-sectional Survey

**DOI:** 10.5681/joddd.2011.013

**Published:** 2011-06-14

**Authors:** Adriane Kamulegeya, Buwembo William, Charles Mugisha Rwenyonyi

**Affiliations:** ^1^ Oral & Maxillofacial Unit, Department of Dentistry, Mulago Hospital, Kampala, Uganda; ^2^ Department of Anatomy, Makerere University College of Health Sciences, Kampala, Uganda; ^3^ Department of Dentistry, Makerere University College of Health Sciences, Kampala, Uganda

**Keywords:** Antibiotic use, dental practitioner, prescription, prophylaxis, Uganda

## Abstract

**Background and aims:**

Irrational prescription of antibiotics by clinicians might lead to drug resistance. Clinicians do prescribe antibiotics for either prophylactic or therapeutic reasons. The decision of when and what to prescribe leaves room for misuse and therefore it is imperative to continuously monitor knowledge and pattern of prescription. The aim of the present study was to determine the knowledge of antibiotic use and the prescription pattern among dental health care practitioners in Uganda.

**Materials and methods:**

A structured and pretested questionnaire was sent to 350 dental health care practitioners by post or physical delivery. All the questionnaires were sent with self-addressed and prepaid postage envelopes to enable re-spondents to mail back the filled questionnaires. Chi-squared test was used to test for any significant differences between groups of respondents based on qualitative variables.

**Results:**

The response rate was 40.3% (n=140). Of these 52.9 % were public health dental officers (PHDOs) and 47.1% were dental surgeons. The males constituted 74.3% of the respondents. There were statistically significant differences be-tween dental surgeons and (PHDOs) in knowledge on prophylactic antibiotic use (P = 0.001) and patient influence on pre-scription (P = 0.001). Amoxicillin, in combination with metronidazole, was the most common combination of antibiotics used followed by co-trimoxazole with metronidazole.

**Conclusion:**

The knowledge of dental health care practitioners in antibiotic use in this study was generally low. A combi-nation of amoxicillin with metronidazole was the most commonly prescribed antibiotics subsequent to different dental pro-cedures.

## Introduction

Management of oral infections represents a big portion of the work handled by oral health care providers; therefore, time and again, they have to prescribe antibiotics to contain or prevent infections. These infections could either be dental, periodontal or originate from any changes that alter the balance between endogenous bacteria and host defense mechanisms. Although a number of studies on antibiotic use have been carried out, controversies still exist in areas such as prophylaxis, interactions with contraceptives, indications in medically compromised patients and their use after both minor and intermediate oral surgical procedures.^1-6^ These controversies lead to inappropriate and unwarranted antibiotic use in human and veterinary medicine,. This misuse contributes to development of specific antibiotic-resistant strains.^7^ Bacteria in most oral infections are indigenous to the oral cavity and the effective antibiotics against them are known. However, reports of emerging resistant strains to these drugs are worrisome.^8,9^ One of the major causes of this emerging resistance is inappropriate use of antibiotics. This leads to selection and dominance of resistant microorganisms, which could transfer resistance genes from antibiotic-resistant to susceptible microorganisms.^9^

Improper antibiotic use includes too low a dose, too long a duration, wrong choice of antibiotics, improper combination of antibiotics and therapeutic or prophylactic use in unwarranted/unproven clinical situations.^8-10^ The emergence of resistant strains is not only dangerous to the affected individual but also has serious public health implications. When the resistant strains affect the community, there is an added health care cost of changing to more expensive antibiotics.^11^

One way of preventing development of antibiotic resistant strains is by the rational use of these drugs. However, this can only be possible if the health care providers are aware and adhere to available antibiotic prescription guidelines.^1-3,8,12,13^ Additionally, they should be updated of emerging resistant strains. Despite the existence of guidelines, there are differences in levels of knowledge and approach to antibiotic prescription among professional oral health care providers.^1-3,8,14^

For instance, although rifampicin is the only antibiotic proven to interfere with contraceptive efficacy, 61.5% of Maltese dentists warned women of child-bearing age of the potential interactions of penicillin-based drugs with oral contraceptives. However, 30.8% of them never cautioned patients of the potential teratogenic effects of metronidazole.^2^ In Kuwait, despite the fact that over 80% of dentists did not have any additional postgraduate training on antibiotic therapy, higher knowledge regarding adequate indications for antibiotic use was associated with longer professional experience.^15^ In a Norwegian study,^16^ it was found that 20% of the dentists did not know that amoxicillin is a penicillin-based drug. We did not come across any published studies on antibiotic prescription pattern of oral health care providers in Uganda. The aim of the present study was to determine the knowledge of antibiotic use and prescription pattern among oral health providers in Uganda.

## Materials and Methods

This was a descriptive cross-sectional study.

### Study population

The study sample consisted of registered oral health care practitioners (n = 350) working in different parts of Uganda. These practitioners included dental surgeons and public health dental officers (PHDOs). The PHDOs are the equivalent of dental therapists/hygienists elsewhere. They are supposed to work under the supervision of dental surgeons, but due to limited financial resources and low numbers of dental surgeons, the PHDOs most times provide oral health care, including antibiotic prescription without supervision.

### Survey tool (questionnaire)

A structured questionnaire was designed, pre-tested and then physically delivered or posted along with a self-addressed prepaid postage envelope to all the registered and practicing dental surgeons and PHDOs in Uganda (see Additional file 1). The respondents were requested to mail back the filled questionnaires within 2 months of receipt. The questionnaire included socio-demographic factors and questions that elicited responses regarding antibiotic prescriptions. The information was kept confidential by eliminating all the possible personal identifiers. The knowledge of respondents was evaluated based on recommended guidelines and standards.^1,9,13,14,18^

### Data analysis

Data was recorded in a computer spread sheet (Microsoft Excel, version 2007, Corp.) and analyzed using Statistical Package for Social Sciences Inc. (version 15 for Windows, Chicago Illinois, USA.). Chi-squared test was used for any significant differences between respondents based on qualitative variables. The time since the respondent last attended a continuing professional development (CPD) course on antibiotic use was categorized as ≤2 years and >2 years. The time since the respondent started dental practice was categorized as: group A: ≤ 5 years; B: 6-10 years; C: 11-15 years; and D: ≥ 16 years. The level of significance was set at 5%.

## Results

Of the 350 survey questionnaires mailed, 140 (40.3%) were returned, of which 52.9 % (n = 74) were from PHDOs and 47.1% (n = 66) from dental surgeons. One hundred four (74.3%) of the respondents were male and 21.4% (n = 30) were female. Six of the respondents did not specify their sex. The proportions of respondents in groups A, B, C and D based on time in dental practice were: 52.9%, 17.1%, 18.6%, and 10.0%, respectively.

Ninety-four (67.1%) of the respondents had attended a CPD course on antibiotic use in the last one year, 8.6% at least once in the last two years, and 7.1% in the last five years while 4.3% reported not having had any since commencement of dental practice. The most common source of the CPD was from drug sales representatives with 28.6%; self-directed learning with 25.7%; conferences with 14.3%; journal clubs with 5.7%; dental schools with 4.3%; hospitals/clinics with 1.4% and a combination of the above with 10.0%. The respondents who were working in public health facilities were 40%; private, 37.1%; training institutions, 12.9%; nongovernmental (NGO) not-for-profit health facilities, 5.7%; a combination of public and private, 2.9%; and public and NGO, 1.4%. The PHDOs and the female respondents were significantly more influenced by the patients into prescribing antibiotics than their respective counterparts (P = 0.001, Table 1). Seventy-six (54.3%) of the respondents gave at least one correct guideline on the use of antibiotics in pregnancy while 35.7% gave inappropriate ones. Antibiotic guidelines for lactating mothers also yielded several responses with 54.3% considered appropriate, 21.4% inappropriate and the rest with no response to the question. Over seventy-two percent of the respondents did not have any information regarding potential antibiotic-contraceptive interaction. The knowledge on antibiotic prophylactic use was significantly higher among dental surgeons (P = 0.001) and in private practice (P = 0.005) as compared to their respective counterparts (Table 1). Forty percent of the respondents gave appropriate indication for prophylactic antibiotic use in dentistry while 44.3% were not considered right. The rest did not reply the question. Knowledge on indications for culture and sensitivity testing was correctly replied by 60% of the respondents while 25.7% did not have any idea. Only 1.4% of the respondents reported the right guidelines in deciding which effective antibiotics to prescribe.

**Table 1:** Frequency distribution of respondents according to responses on use of antibiotics (n = 140)

Variable	Response	Category	Prevalence (%)	P
Knowledge on prophylactic use of antibiotics	Correct	Dentist	69.2	0.001
		PHDO	31.3	
		Public	33.3	0.005
		Private	57.1	
Patients' influence on antibiotic prescription	Strong to very strong	Male	27.6	0.001
		Female	66.7	
		Dentists	31.3	0.001
		PHDO	72.2	
Indications for culture and sensitivity	Correct	Public	44.4	0.02
		Private	69.2	
Use of systemic antibiotics in dry socket	Yes	≤2 years of last antibiotic course	66.0	0.01
		>2 years of last antibiotic course	50.0	
Antibiotic use in endodontic therapy	Always and frequently	≤5 years of graduation	49.0	0.001
		>5 years of graduation	33.3	

*Therapeutic and prophylactic use of antibiotics*

**Figure 1:**
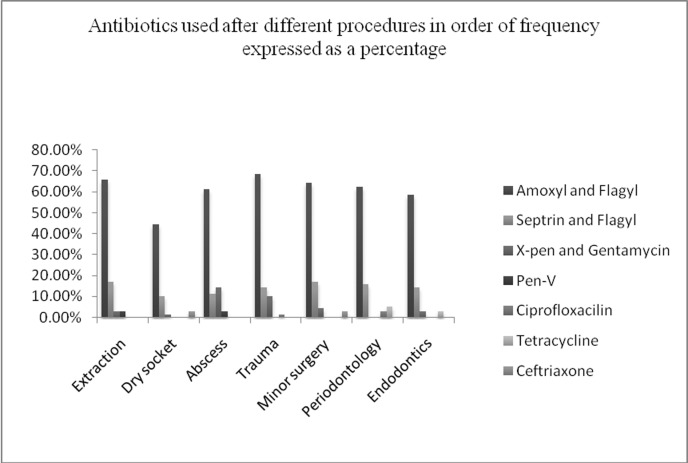
Antibiotics used after different procedures in order of frequency expressed a percentage

**Figure 2:**
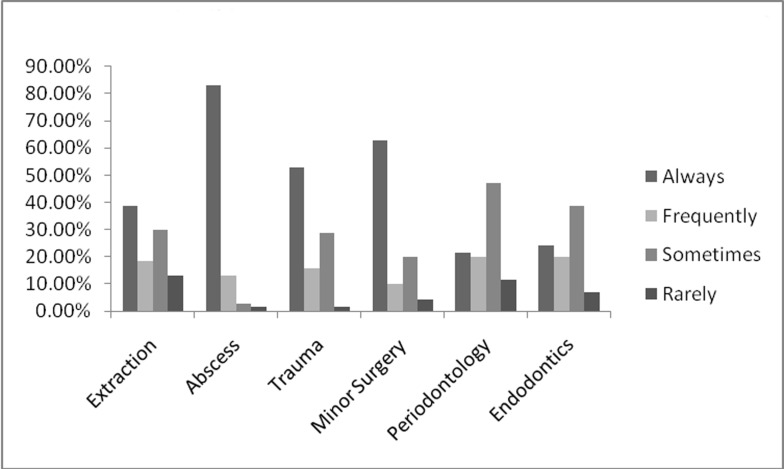
Antibiotic prescription frequency expressed as percentage of total respondents following different dental procedures

The antibiotic predominantly prescribed after any form of treatment either as a single drug or in combination was amoxicillin. The combination of amoxicillin together with metronidazole was most frequently prescribed followed by co-trimoxazole and metronidazole, crystalline penicillin and gentamicin.Figure 1 shows the four most commonly used antibiotics subsequent to different dental procedures. The respondents were also asked on the frequency of antibiotic prescription after specific dental treatment and the results are shown in Figure 2.

All the respondents mentioned amoxicillin and metronidazole as their drugs of choice for prophylactic use. There was no respondent who mentioned the alternative drug(s) in patients allergic to specific antibiotics.

## Discussion

Many factors have been reported to influence antibiotic prescription pattern among dental health care practitioners. These range from culture, patient preferences, treatment methods, prevalence of disease, available resources, payment systems, education background, and the existence and application of clinical guidelines.^15,16,19,20^ In the present study, 67.1% of the respondents had some form of continuing professional development on the use of antibiotics in the last one year, particularly from drug sales representatives. This is not an appropriate source of CPD since the priorities of the drug sales representatives are about sales volumes. The drug information they give may not be evidence-based, additionally they may recommend antibiotics of questionable efficancy.^21^

Training background in dentistry has been reported to have an influence on antibiotic prescription.^14^ The PHDOs were significantly more influenced by the patients into prescribing antibiotics during treatment as compared to dental surgeons (Table 1). In Uganda, the dental surgeons and PHDOs are trained under different curricula and by trainers with different qualifications, which could partly explain the observed difference in patients’ influence. The female dental health care practitioners were also significantly more influenced by patients into prescribing antibiotics as compared to their male counterparts (Table 1). The gender difference in the prescription of drugs has been reported by other authors,^22^ but the reasons for the observed trend are not obvious.

Approximately 54.3% of the respondents gave a correct response on the knowledge about antibiotic use for expectant and lactating mothers. Some of the wrong answers given by the respondents included: increased risk of infection during pregnancy, pregnancy as a contraindication for dental treatment while some singled out wrong antibiotics such as amoxicillin as the cause of stained teeth among children when given to lactating mothers.

In the present study, 72.9% of respondents did not have any knowledge regarding potential antibiotic-contraceptive interaction. This finding was very different from Montazem,^2^ who reported that 61.5% of Maltese dentists warned female patients of the potential interaction. Although the veracity of this information, except for rifampicin, is still controversial,^4,5^ the fact that such a high percentage of the respondents had not heard of it is a source of concern.

The indications for antibiotic prophylaxis in dentistry are not etched in stone due to frequent updates and changes in guidelines,^2,12,23,24^ In the present study, 44.4% of the respondents gave wrong indications for prophylactic use of antibiotics. This finding confirms previous reports.^17,25^ The wrong indications are further complicated by anecdotal reports of infection after dental extractions.^26^ It is also worth noting that although diabetes mellitus was taken as a correct answer very few respondents listed non-/poorly-controlled diabetes mellitus as their indication. This suggests that there is need to instruct oral health care providers in specific prevailing conditions that warrant prophylactic antibiotic use.^19,23,24^

A combination of amoxicillin with metronidazole was the most commonly prescribed antibiotics subsequent to different dental procedures (Figure 1). However, most reports^15,16,20,25,27,28^ on antibiotic use in dentistry show amoxicillin or other penicillin-based drugs to be the most commonly used. This trend is based on the established efficacy of penicillin-based drugs on bacteria involved in odontogenic infections.^8,29,30^ Increasingly resistant strains are being reported in odontogenic infections,^31,32^ hence the need for constant antibiotic pharmacovigilance. Similar to a Nigerian study,^33^ our respondents gave co-trimoxazole as one of their antibiotics of choice. This is a cause for concern since over-consumption of co-trimoxazole and emerging resistance of organism for which it is used as a prophylactic agent has been reported.^34^ In corroboration to previous reports,^27,28,33^ our respondents revealed very frequent use of metronidazole alone or in combination with other antibiotics (Figure 1). In fact at one time in Britain, metronidazole accounted for the highest number of antibiotic prescriptions by dentists.^35^

In the present study up to 38% of respondents always prescribed systemic antibiotics following routine extraction (Figure 2). Previous studies,^17,36^ have recommended antibiotic prescription after extraction in the presence of relevant medical history and localized or generalized swellings. Although a high level of bacteremia follows tooth extraction compared to other oral surgical procedures,^37^ the routine use of antibiotics is not warranted.

In our study, over 40% of the respondents routinely or frequently prescribed systemic antibiotics in periodontal therapy. However, 55.7% of the respondents gave correct indication for their use. Although systemic antibiotic therapy can provide great benefit to periodontal patients who do not respond to mechanical periodontal therapy and those with acute periodontal infections associated with systemic medical conditions, its routine use is not recommended.^37^ Amoxicillin in combination with metronidazole was the overwhelming choice of antibiotic by most respondents (Figure 1). Amoxicillin, which is a newer generation penicillin, has the same gram-positive spectrum as benzylpenicillin, in addition to gram-negative spectrum. However, there is increasing evidence that quite a number of gram-negative organisms are developing resistance to it.^31,32^ The frequent use of amoxicillin may have a role in the emerging resistance.

## Conclusion

The present study showed that although the majority of the dental health care practitioners in Uganda had been exposed to CPD on antibiotic use during the previous one year, their knowledge on antibiotic use was generally low. A combination of amoxicillin with metronidazole was the most commonly prescribed antibiotics subsequent to different dental procedures. Sensitivity tests to the most commonly used antibiotics should routinely be carried out to establish drug effectiveness and establish any emerging resistance.

Additional file 1. Antibiotic use in dentistry in Ugandan (Self-administered questionnaire). This material (Word 97-2003 Document) is available online.

## References

[1] Tong DC, Rothwell BR (2000). Antibiotic prophylaxis in dentistry: a review and practice recommendations. J Am Dent Assoc.

[2] Montazem A (1998). Antibiotic prophylaxis in dentistry. Mt Sinai J Med.

[3] Poeschl PW, Eckel D, Poeschl E (2004). Postoperative prophylactic antibiotic treatment in third molar surgery — a necessity?. J Oral Maxillofac Surg.

[4] Termine N, Panzerella V, Ciavarella D, Lo Muzio, D’Angelo M, Sardella A, et al (2009). Antibiotic prophylaxis in dentistry and oral surgery: use and misuse. Int Dent J.

[5] Wahl MJ. Demystifying medical complexities. J Calif Dent Assoc 2000;28:510–8. Accessed 2009 Nov 20. Available from: http://www.cda.org/library/cda_member/pubs/journal/ jour0700/complex.html 11324133

[6] Esposito M, Coulthard P, Oliver R, Thomsen P, Worthington HV. Antibiotics to prevent complications following dental implant treatment. Cochrane Database of Systematic Reviews 2003; 3:CD004152. DOI: 10.1002/14651858. CD004152. 10.1002/14651858.CD004152 12918006

[7] van de, Grundmann H, Verloo D, Tiemersma E, Monen J, Goossens H, Ferech M (2008). Antimicrobial drug use and resistance in Europe. Emerg Infect Dis.

[8] Peterson LJ (1987). Antibiotics: their use in therapy and prophylaxis. In: Topazian RG, Goldenberg MH, eds. Management of Infections of the Oral and Maxillofacial Region.

[9] American Dental, Council on (1997). Antibiotic use in dentistry. J Am Dent Assoc.

[10] Tenover FC, Hughes JM (1996). The challenges of emerging infectious diseases. J Am Med Assoc.

[11] Cranny G, Elliott R, Weatherly H, Chambers D, Hawkins N, Myers L, et al. A systematic review and economic model of switching from non-glycopeptide to glycopeptide antibiotic prophylaxis for surgery. Health Technol Assess 2008;12: iii-iv, xi-xii, 1-147. 10.3310/hta12010 18093447

[12] Ashrafian H, Bogle RG (2007). Antimicrobial prophylaxis for endocarditis: emotion or science?. Heart.

[13] Council on Clinical Affairs. Guidelines on appropriate use of antibiotic therapy for pediatric dental patients. In: American Academy of Pediatric Dentistry Reference Manual. American Academy of Pediatric Dentistry; 2010;29:246-8. Available from: http://www.aapd.org/media/policies_ guidelines/g_antibiotictherapy.pdf

[14] Yehuda Z, Liran L (2008). Clinical decision making in restorative dentistry, endodontics, and antibiotic prescription. J Dent Educ.

[15] Salako NO, Rotimi VO, Adib SM, Al-Mutawa S (2004). Pattern of antibiotic prescription in the management of oral diseases among dentists in Kuwait. J Dent.

[16] Demirbas F, Gjermo P, Preus HR (2006). Antibiotic prescribing practices among Norwegian dentists. Acta Odontol Scand.

[17] Epstein J, Chong S, Nhud. LE (2000). A survey of antibiotic use in dentistry. J Am Dent Assoc.

[18] Suresh L, Radfar L (2004). Pregnancy and lactation. Oral Surg Oral Med Oral Pathol Oral Radiol Endod.

[19] Fine HD, Hammond FB, Loesche JW (1998). Clinical use of antibiotics in dental practice. Int J Antimicrob Agents.

[20] Dailey YM, Martin VM (2001). Are antibiotics being used appropriately for emergency dental treatment?. Br Dent J.

[21] Lexchin J (1997). What information do physicians receive from pharmaceutical representatives?. Can Fam Physician.

[22] Sequeira RP, Al Khaja, Damanhori AH, Mathur VS (2003). Physician gender and antihypertensive prescription pattern in primary care. J Eval Clin Pract.

[23] Wilson W, Taubert KA, Gewitz M, Lockhart PB, Baddour LM, Levison M, et al. Prevention of infective endocarditis: guidelines from the American Heart Association: a guideline from the American Heart Association Rheumatic Fever, Endocarditis and Kawasaki Disease Committee, Council on Cardiovascular Disease in the Young, and the Council on Clinical Cardiology, Council on Cardiovascular Surgery and Anesthesia, and the Quality of Care and Outcomes Research Interdisciplinary Working Group. J Am Dent Assoc 2007;138:739-45, 747-60. 10.14219/jada.archive.2007.0262 17545263

[24] Canadian Dental Association. CDA position on antibiotic prophylaxis for dental patients at risk. Accessed 2010 Jan 3. Available form: http://www.cda-adc.ca/_files/position_ statements/antiobiotic_prophylaxis_joint.pdf

[25] Palmer NA, Pealing R, Ireland RS, Martin MV (2000). A study of prophylactic antibiotic prescribing in National Health Service general dental practice in England. Br Dent J.

[26] Fe Marqués, Maestre Vera, Mateo Maestre, González Romo, Castrillo Amores (2008). Septic arthritis of the knee due to Prevotella loescheii following tooth extraction. Med Oral Patol Oral Cir Bucal.

[27] Jaunay T, Sambrook P, Goss A (2000). Antibiotic prescribing practices by South Australian general dental practitioners. Austral Dent J.

[28] Dar-Odeh NS, Abu-Hammad OA, Khraisat AS, El Maaytah, Shehabi A (2008). An analysis of therapeutic, adult antibiotic prescriptions issued by dental practitioners in Jordan. Chemotherapy.

[29] Poveda-Roda R, Bagán JV, Sanchis-Bielsa JM, Carbonell-Pastor E. Antibiotic use in dental practice. A review. Med Oral Patol Oral Cir Bucal 2007;12:E186–92. 17468711

[30] Sweeney LC, Dave J, Chambers PA, Heritage J (2004). Antibiotic resistance in general dental practice—a cause for concern?. J Antimicrob Chemother.

[31] Ready D, Lancaster H, Qureshi F, Bedi R, Mullany P, Wilson M (2004). Effect of amoxicillin use on oral microbiota in young children. Antimicrob Agents Chemother.

[32] Kuriyama T, Nakagawa K, Karasawa T, Saiki Y, Yamamoto E, Nakamura S (2000). Past administration of beta-lactam antibiotics and increase in the emergence of beta-lactamase-producing bacteria in patients with orofacial odontogenic infections. Oral Surg Oral Med Oral Pathol Oral Radiol Endod.

[33] Ogunbodede EO, Fatusi AO, Folayan MO, Olayiwola G (2005). Retrospective survey of antibiotic prescriptions in dentistry. J Contemp Dent Pract.

[34] Grimwade K, Gilks C (2001). Cotrimoxazole prophylaxis in adults infected with HIV in low-income countries. Curr Opin Infect Dis.

[35] Al-Haroni M (2008). Bacterial resistance and the dental professionals’ role to halt the problem. J Dent.

[36] Palmer NO, Batchelor PA (2004). An audit of antibiotic prescribing by vocational dental practitioners. Prim Dent Care.

[37] Takai S, Kuriyama T, Yanagisawa M, Nakagawa K, Karasawa T (2005). Incidence and bacteriology of bacteremia associated with various oral and maxillofacial surgical procedures. Oral Surg Oral Med Oral Pathol Oral Radiol Endod.

